# Right upper quadrant pain: a case where diagnosis was made from the chest X-Ray

**DOI:** 10.11604/pamj.2014.17.60.3494

**Published:** 2014-01-26

**Authors:** Theocharis Koufakis, Anastasios Margaritis

**Affiliations:** 1 General Hospital of Larissa, Department of Internal Medicine, Larissa, Greece

**Keywords:** Chest X-Ray, hydatid cyst, lung

## Image in medicine

A 90 years old woman, in good physical condition, and without history of chronic disease, smoking or alcohol abuse, presented to the Emergency Department of our Hospital, complaining about abdominal pain with a specific location at the right upper quadrant. Her symptoms started approximately a month ago. She was a farmer and a habitant of a Greek rural district. Her blood tests were all within the normal ranges, but her chest x-ray revealed a surprise at the right lower: a big, spherical cystic lesion with a characteristic calcified ring around it, coming out of the right lung. In view of this finding, the patient was admitted and abdominal computer tomography (CT) and ultrasound were performed, which demonstrated the typical imaging features of an hydatid cyst (8.5 x 9.5 cm), sited at the right lobe of the liver. Surgical treatment of the cyst was not preferred, because of the age of the patient and the possibility of postoperative complications. In her follow up, she remained in good health and free of symptoms. The differential diagnosis of liver hydatid cyst includes polycystic liver disease, hepatic abscess, hepatocellular carcinoma, hepatic and amebic cysts, but the very specific imaging findings in CT are usually enough to establish the diagnosis. In conclusion, this case underlines the diagnostic value of the chest x-ray which remains the keystone imaging method for any clinical physician. Furthermore, echinococcosis, although considered as a “forgotten” disease, still remains a public health problem for many epidemic areas around the world.

**Figure 1:**
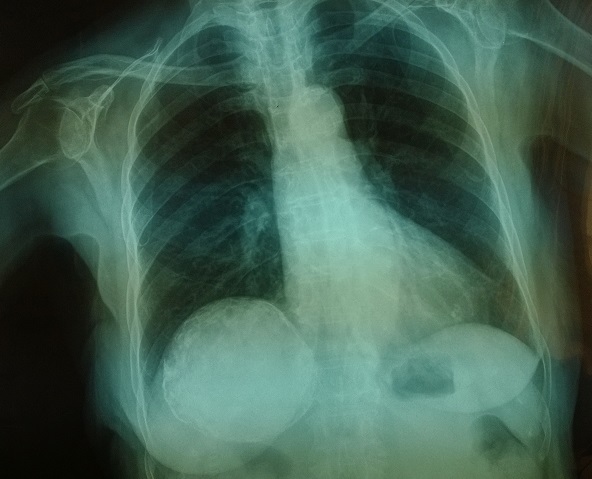
A large hydatid cyst coming from the right lobe of the liver with the characteristic calcified ring around it

